# The laccase gene *MdLac18* decouples the growth-defense trade-off by orchestrating lignification to enhance aphid resistance and biomass accumulation in *Nicotiana benthamiana*

**DOI:** 10.3389/fpls.2026.1799585

**Published:** 2026-04-14

**Authors:** Jinyang Li, Mengxia Cui, Ziwen Teng, Fanglong Chen, Yinjun Fan, Ying Wang, Xuechao Zhang, Shengjun Zhang, Fanghao Wan, Hongxu Zhou

**Affiliations:** 1 College of Plant Health and Medicine, China-Australia Cooperative Research Center for Crop Health and Biological Invasions, Fruit Tree Germplasm Innovation and Green Production Research and Development Team, Qingdao Agricultural University, Qingdao, China; 2 Agro-Tech Extension and Service Center of Fengxiang District, Baoji, Shaanxi, China; 3 Institute of Agricultural Sciences of Ili Kazakh Autonomous Prefecture, Yining, China; 4 Agricultural Genomics Institute at Shenzhen, Chinese Academy of Agricultural Sciences, Shenzhen, Guangdong, China

**Keywords:** growth-defense trade-off, insect resistance, laccase, lignin, transgenic plants

## Abstract

Enhancing resistance to piercing-sucking pests while preserving superior agronomic performance remains a key challenge in plant breeding, constrained by the classical “growth-defense trade-off”. Lignin, a core component of plant secondary cell walls, acts as a vital physical barrier against pest invasion, yet genetic regulators that simultaneously reinforce lignin-mediated defense and promote plant growth are rarely reported. Here, we cloned *MdLac18* (GenBank Accession No.: PV341664), a laccase gene from the aphid-resistant apple cultivar ‘Starkrimson’, and heterologously expressed it in *Nicotiana benthamiana* via Agrobacterium-mediated transformation. Transgenic lines exhibited robust resistance to *Myzus persicae*: corrected aphid mortality reached 43.99% and fecundity decreased by 55.13% at 8 days post-inoculation. Electrical Penetration Graph (EPG) analysis revealed prolonged salivation (E1 wave) and shortened phloem ingestion (E2 wave) in aphids, reflecting impaired stylet penetration. *MdLac18* overexpression increased laccase activity by 59.61% and lignin content by 47.40%, with enhanced vascular tissue lignification. GC-MS analysis confirmed a 176% increase in G-type lignin monomers (coniferyl alcohol derivatives), indicating specific promotion of G-type lignin biosynthesis. Notably, unlike typical defense-related genes, *MdLac18* conferred dual benefits: transgenic tobacco showed improved agronomic traits (increased plant height, stem diameter at the early vegetative stage (30–60 days after transplantation), biomass, and early flowering). Our findings establish *MdLac18* as a rare genetic resource that, by exerting its stress-regulatory function and enhancing plant adaptability to adverse environments, holds the potential to decouple the growth-defense trade-off under controlled suboptimal conditions, thus providing a novel strategy for breeding crops with durable aphid resistance and superior agronomic performance.

## 1 Introduction

China dominates global apple production, contributing over 80% of the world’s total output and serving as the primary driver of the global apple industry (Zhou et al., 2015). However, this vital sector faces severe threats from aphid species, particularly *Myzus persicae* (green peach aphid) and *Eriosoma lanigerum* (woolly apple aphid). *M. persicae*, a piercing-sucking pest, is a worldwide agricultural pest with a broad host range, capable of infesting over 300 crop species including apples and tobacco. It pierces and sucks phloem sap from young leaves and shoots of plants, causing leaf yellowing, curling and crinkling, impairing photosynthesis, and even leading to defoliation in severe cases. The honeydew it secretes induces sooty mold, and it also transmits viral diseases, which significantly reduce crop yield and quality and result in enormous economic losses (Bass et al., 2014; Olympia et al., 2006). Current pest management relies heavily on chemical pesticides, leading to environmental pollution, pesticide resistance, and food safety concerns (Guo et al., 2022). Thus, developing genetically improved crops with intrinsic aphid resistance is an urgent and sustainable solution for agricultural development.

To counter such herbivorous threats, plants have evolved sophisticated defense mechanisms against insect attack, with lignin biosynthesis via the phenylpropane pathway emerging as a key defensive strategy (An et al., 2019; Ma, 2024; Choung et al., 2025). As a major component of the secondary cell wall, lignin forms a rigid physical barrier that obstructs the penetration of insect stylets, thereby limiting pest feeding (Wang et al., 2020; Xie et al., 2020). Angiosperm lignin is composed of three monomeric units: guaiacyl (G), syringoyl (S), and p-hydroxyphenyl (H) lignin, whose polymerization is tightly regulated by a cascade of enzymes (Xie et al., 2020). Within the lignin biosynthesis pathway, laccase (LAC)—a multicopper oxidase—acts as the rate-limiting enzyme in the final step of lignin polymerization catalyzing the oxidation of monolignols (coniferyl alcohol, sinapyl alcohol, and p-coumaroyl alcohol) into G, S, and H lignin monomers, respectively (Ishida et al., 2024; Qin et al., 2020; Zhuo et al., 2024). Accumulating evidence highlights its critical role in plant resistance to pathogens and insects. For instance, overexpression of cotton *GhLac1* enhances lignin accumulation to confer resistance against cotton bollworm and cotton aphid (Hu et al., 2018), while wheat *TaLAC4* improves resistance to *Fusarium graminearum* by promoting G-type lignin synthesis and cell wall thickening (Nancy et al., 2020). Similarly, cotton *GhLAC15*, *GhLAC4A* and *GhLAC14–3* positively regulate *Verticillium wilt* resistance through defense-induced lignification (Zhang et al., 2019; Wei et al., 2021; Cheng et al., 2025). However, the role of laccase genes from woody plants in mediating aphid resistance remains largely unexplored.

Furthermore, most studies on laccase-mediated resistance have focused on model plants (e.g., *Arabidopsis thaliana*, *Oryza sativa*) or disease resistance, with limited exploration of their function in aphid resistance—especially in perennial woody crops like apple (*Malus domestica*). Apple possesses unique advantages in lignin metabolism, including high lignin demand, diverse laccase gene families, and complex lignin structures, which endow it with durable insect resistance advantages rarely found in herbaceous plants (Qin et al., 2020; Liu et al., 2024). Our recent transcriptomic and proteomic analyses identified 27 differentially expressed laccase genes in the aphid-resistant apple cultivar ‘Starkrimson’ following *E. lanigerum* infestation, suggesting their potential involvement in aphid defense (Li et al., 2023). Nevertheless, the specific aphid-resistant function and underlying mechanism of apple-derived laccase genes remain largely unexplored—especially whether these woody plant-specific laccase genes can mediate aphid resistance through lignin regulation, as reported in herbaceous crops (Hu et al., 2018; Nancy et al., 2020).

A major bottleneck in plant breeding is the “growth-defense trade-off”—a classical paradigm wherein plants allocate metabolic resources to defense at the cost of vegetative growth and yield (Huot et al., 2014; Yang et al., 2024). Lignin biosynthesis and secondary metabolism consume substantial energy and carbon skeletons, which would otherwise support plant growth and reproduction (Huot et al., 2014). Thus, identifying genetic factors that can decouple growth and defense—i.e., enhancing resistance without compromising agronomic traits—represents a significant breakthrough in crop improvement (Yang et al., 2024). Although some laccase genes have been reported to regulate plant growth and development (Qin et al., 2020), no apple laccase gene has been demonstrated to simultaneously enhance aphid resistance and promote plant growth—representing a critical gap in utilizing woody plant genetic resources to break the growth-defense trade-off for crop improvement (Yang et al., 2024).

To address these knowledge gaps, we selected three apple cultivars with distinct aphid resistance levels ([‘Fuji’ - susceptible, ‘Ralls Genet’- resistant, and ‘Starkrimson’ - highly resistant]) for cross-cultivar comparison. Our recent transcriptomic and proteomic analyses identified 27 differentially expressed laccase genes in the aphid-resistant apple cultivars following *E. lanigerum* infestation (Li et al., 2023), and we selected some of these differentially expressed genes for functional validation via transgenic expression in tobacco, which aimed to: (1) validate the aphid-resistant function of apple laccase gene in transgenic *N. benthamiana* by assessing aphid survival, fecundity, and feeding behavior; (2) elucidate the underlying mechanism by quantifying laccase activity, lignin content, combined with histological analysis of cell wall lignification; (3) evaluate the effect of apple laccase gene overexpression on key agronomic traits (e.g., plant height, stem diameter, biomass, and flowering time) to verify its potential in breaking the growth-defense trade-off. Fortunately, we found that MdLac18 (GenBank Accession No.: PV341664, from the aphid-resistant apple cultivar ‘Starkrimson’) not only confers aphid resistance but also promotes tobacco growth. In our previous transcriptome analysis (Li et al., 2023), MdLac18 was temporarily named LAC1 during preliminary screening of differentially expressed genes. In this study, we performed a standardized nomenclature of all laccase genes in apple, and this gene (ID: MD03G1106500) was formally renamed MdLac18 according to its phylogenetic relationship, chromosomal location, and domain structure. We hypothesize that *MdLac18* enhances aphid resistance by promoting lignin accumulation and cell wall lignification, thereby obstructing aphid stylet penetration; meanwhile, it improves plant growth performance through enhanced structural support (e.g., reinforced xylem vessels) or auxin-mediated cell elongation (rather than lignin conductivity), thus breaking the growth-defense trade-off. The findings of this study will not only identify a novel apple-derived laccase gene with dual functions in aphid resistance and growth promotion but also provide a technical strategy for utilizing woody plant genetic resources in crop improvement, ultimately contributing to reduced chemical pesticide use and enhanced sustainability in agricultural systems.

## 2 Materials and methods

### 2.1 Experimental materials

The sterile *N. benthamiana* plants were cultured at room temperature of 24 ± 2°C, relative humidity of 50 ± 10% and photoperiod (light:dark) of 16:8. The experimental *M. persicae* was raised in an intelligent artificial climate box with the relative humidity of 60 ± 10% and photoperiod (light: dark) of 16:8 at 25 ± 2°C, and the soil-cultured tobacco seedlings obtained from the seed sowing and growth of *N. benthamiana* were used for reproduction.

The silver colloid was donated by Institute of Chemical Ecology, College of Plant Protection, Henan Agricultural University, and other conventional reagents were purchased from Keshang Biological Co., Ltd.

### 2.2 Experimental methods

#### 2.2.1 Screening, cloning and phylogenetic evolution of LAC gene from apple

Three Starkrimson plants damaged by woolly apple aphids and with consistent growth were selected. The second spreading leaf with the tip downwards from each plant was cut under aseptic operation and stored at 4°C for later use. The total RNA was extracted from the leaves using an RNA extraction kit and synthesized into cDNA by reverse transcription. With reference to the sequences of the target gene and the vector, homologous recombination primers were designed through the Vazyme single fragment cloning website (**Table 1**). EGFP was used as the technical control. Using the cDNA as a template and the primers listed in **Table 1**, PCR amplification was performed using 2×Phanta Max Master Mix(Dye Plus) reagent, and the annealing temperature was 58°C. The obtained products were retained for subsequent downstream experiments.

**Table 1:** Primers used for gene cloning with homologous arms.

Primer name	Sequence (5’-3’)	Length/bp
MdLac18-P-F	gagaacacgggggactctagaATGGGAGTTCCTCTTCTTTCGTC	1792
MdLac18-P-R	cgatcggggaaattcgagctcTTAACATGTGGGAAGATCTGCTGG	
EGFP-P-F	ggactctagaggatccccgggATGGTGAGCAAGGGCGAGG	760
EGFP-P-R	cgatcggggaaattcgagctcTTACTTGTACAGCTCGTCCATGCC	
PBI121-5570-5590-F	TGCCATCATTGCGATAAAGGA	2356
PBI121-7946-7926-R	ATTTATCCTAGTTTGCGCGCT	

The amino acid sequences of MdLac18 and 17 laccase genes from Arabidopsis thaliana were aligned using ClustalW. A phylogenetic tree was constructed using the Neighbor-Joining (NJ) method in MEGA X. The reliability of the tree branches was assessed by bootstrap analysis with 1000 replicates.

#### 2.2.2 Construction of plant overexpression vector

The pBI121 plasmid was linearized by double restriction endonuclease digestion of QuickCut™ Xba I and QuickCut™ Sac I, and the recovered restriction endonuclease product was purified.

The PCR purified product of the target gene was connected to the linearized vector mentioned above according to the instructions of the one-step cloning kit of ClonExpress^®^ II. The ligation product was converted to DH5α competent cells by heat shock. The PCR system was prepared and amplified with reference to 2 × Taq Plus Master Mix II (Dye Plus), and the positive clones were screened by gel electrophoresis detection and sequenced.

#### 2.2.3 Agrobacterium-mediated tobacco leaf disc genetic transformation

The positive clones with correct sequencing were shaken for culture, followed by plasmid extraction and transformation according to the EHA105 Agrobacterium competence manual. The plates containing Kan and Rif were coated with the conversion solution, and the plates were cultured upside down at 28°C for 48 h. The next day, monoclonal antibody was selected for PCR identification of bacterial solution, and the correct bacterial solution could be directly used for experiment.

The tobacco seeds were successively sterilized with 75% ethanol and 2.5% NaClO for 30 s, rinsed with sterile water for three times, dried, inoculated on MS medium, and cultured under sterile light at 25°C for 4 weeks, to obtain leaves for later use. Agrobacterium tumefaciens OD_600_ was cultured to 0.8–1.2, with reference to the leaf-disc method of Horsch et al. (1985) for agrobacterium tumefaciens.

#### 2.2.4 Identification and screening of transgenic tobacco

The transgenic and control tobacco leaves acclimated for 1 month were taken, and the leaf DNA was extracted with reference to Fast Pure Plant DNA Isolation mini kit. PCR detection was performed using M13 universal primers (**Table 2**), and PCR products were analyzed by 1% agarose gel electrophoresis to screen positive transgenic plants.

**Table 2:** Primers for the identification of transgenic tobacco.

Primer name	Sequence (5’-3’)	Application
q-MdLac18-F	accttctccgcctaatcaacg	Fluorescence quantification
q-MdLac18-R	gctttggtcttgagcagaacatt	
q-EFlα-F	agcttcacctcccaggtcatc	Fluorescence quantification (internal reference)
q-EFlα-R	agaacgcctgtcaatcttgg	
M13-F	CAGGAAACAGCTATGAC	Identification of transgenic plant
M13-R	GTAAAACGACGGCCAGT	

According to DNA identification results, RNA of positive plants was extracted and reverse-transcribed into cDNA; Using tobacco NbEF1α gene as the internal reference, qPCR (three replicates) was performed using the primers listed in **Table 2** to detect the expression level of the target gene in the transgenic plants.

#### 2.2.5 Phenotypic observation of transgenic tobacco

The plants with good growth and high gene expression were selected and cultured in the growth chamber of 25°C, and the plant height, internode length, stem diameter (measured at the stem base with a vernier caliper, accuracy 0.01 mm), flowering time (the complete unfolding of the first flower petal as the judgment standard) and biomass indicators were observed and recorded every 15 days for 90 days. The measurement was repeated for three times to obtain the average, and compared with the non-transgenic wild-type (WT) plants. Statistical analysis for pairwise comparisons were conducted using t-test.

#### 2.2.6 Transgenic tobacco aphid resistance determination

In order to explore the feeding behavior of *M. persicae* on transgenic tobacco, adult aphids were selected as test insects and the plants with high expression of target gene and good growth performance were selected for the determination of aphid resistance function. The Giga-8dd DC-EPG system was used to monitor the probing and feeding behavior of aphids on tobacco leaves. Fifteen replicates per group (1 aphid per plant, single test for 6 h); The valid data were labeled with waveforms (non-probe wave np, intercellular pathway wave C, mechanical injury wave pd, phloem salivation wave E1, phloem persistent feeding wave E2, and xylem feeding wave G) according to Tjallingii (1988) standard by Stylet+a system, and SPSS25.0 software was used for independent sample t-test of EPG data.

The leaves of *N. benthamiana* transgenic with laccase gene (control was normal *N. benthamiana*) were inserted into the culture dish containing 25 mL agar medium, and six second-instar *M. persicae* were inoculated on each leaf, and 15 groups of parallel replicates were set. The survival number and aphid yield of *M. persicae* were recorded for seven consecutive days after inoculation. At the end of the experiment, the log-rank test for survival rate and the independent sample t-test for aphid production were performed with GraphPad Prism 8 software to analyze the effects of tobacco varieties on the survival and reproduction of *M. persicae*.

#### 2.2.7 Transgenic tobacco laccase activity detection

The Solarbio laccase activity detection kit was used for determining the laccase activity of tobacco leaves based on a microplate method. Accurately weighing 0.1 g of laccase gene-transferred tobacco and control tobacco leaves, and adding 1 mL of laccase extracting solution under an ice bath to fully homogenize; Then the samples were centrifuged at 10000 g for 10 min at 4°C and the supernatant was collected for ice bath. The laccase specifically reacted with the ABTS substrate of the kit to generate ABTS free radicals, and the generation rate of ABTS free radicals at the wavelength of 420 nm was dynamically monitored by a microplate reader. Enzyme activity units were defined as the amount of enzyme (U/g) required to oxidize 1 nmol ABTS per minute per gram of sample. Calculated according to the following formula: laccase activity (U/g) = 61.7×ΔA÷W (ΔA is the absorbance difference, and W is the sample mass, calculated according to the manufacturer’s instructions). Independent-samples t-test was performed using GraphPad Prism 8 and the significance of the differences was analyzed.

#### 2.2.8 Determination of lignin content of transgenic tobacco

The total lignin content was determined based on the acetylation method using Suzhou Gledith Lignin Content Determination Kit, with non-transgenic tobacco as the control. The transgenic tobacco leaves with laccase gene and the control leaves with synchronous growth and the same part were selected. After the sample was processed, Reagent 1, Reagent 2 and acetic acid were sequentially added, and after uniform mixing, the absorbance at 280 nm wavelength was detected by using a microplate reader. Referring to the standard curve of the kit, the content was calculated according to the formula: lignin (mg/g) = [(ΔA+0.003)÷10.615]×V1÷W×2 (ΔA is the absorbance difference, V1 is the total volume with constant volume, and W is the sample weight). The data were subjected to independent sample t-test using GraphPad Prism 8 software to analyze the significant difference.

#### 2.2.9 Structural changes of lignin in stems of transgenic tobacco plants

In order to observe the morphological changes of lignin in the cell wall of laccase-transgenic tobacco, phloroglucinol lignin staining kit was used for staining analysis. The transgenic laccase gene tobacco and the non-transgenic control tobacco with consistent physiological states are selected, and stem segments at the same parts are taken to prepare slice samples. Reagents A and B were gradually added dropwise, and the reaction was performed at an interval of 3 min. After the adequate reaction, a cover glass was covered and the samples were observed under the microscope.

#### 2.2.10 Determination of lignin monomers in transgenic tobacco plants

To investigate the composition and content of lignin monomers in tobacco plants expressing the *MdLac18* gene, thioglycolic acid hydrolysis was employed for lignin monomer preparation in this study. Briefly, 5 mg of extract-free lignocellulose samples from tobacco were accurately weighed into 5 mL screw-cap reaction vials. Subsequently, 0.2 mg of the internal standard tetracosane (dissolved in dichloromethane) and 4.0 mL of freshly prepared thioglycolic acid hydrolysis reagent were sequentially added. Immediately after sample addition, the vials were tightly capped and thoroughly vortexed. The reaction mixtures were incubated in a heating mantle at 100 °C for 4 h, with shaking of the vials every 1 h.

After the reaction, the vials were cooled in an ice-water bath for 10 min. The pH of the mixtures was adjusted to 7 using 0.4 mol/L NaHCO3, followed by acidification to pH < 3 with 1 mol/L hydrochloric acid. The resulting mixtures were subjected to conventional liquid-liquid extraction three times with CH2Cl2. After vortexing and phase separation, the lower organic phase was collected and dried over anhydrous MgSO4. The dried organic extracts were evaporated to dryness at 45 °C. The residues were redissolved in 1 mL of, and aliquots of 100 μL were subjected to silylation. The silylation reaction was performed using 100 μL of BSTFA and 20 μL of pyridine at 50 °C for 40 min. The silylated samples were analyzed by gas chromatography-mass spectrometry (GC-MS) to obtain the corresponding total ion chromatograms (TICs). All samples were tested in triplicate. The putative lignin monomers were identified by mass spectral library searching using the NIST MS Search v.2.3 software, and the relative contents of H (p-hydroxyphenyl), G (guaiacyl) and S (syringoyl) lignin monomers as well as the S/G ratio were calculated. Statistical significance of differences was analyzed via one-way ANOVA followed by Tukey’s *post hoc* test (P < 0.05) using GraphPad Prism 8.

## 3 Results and analysis

### 3.1 Gene cloning and phylogenetic evolution

We successfully cloned Starkrimson-*MdLac18* (*MdLac18*) gene. The sequencing results showed that the gene was 1752 bp in length, encoding 583 amino acids, and the predicted molecular weight was 64.418 KDa. Amino acid sequence analysis showed that *MdLac18* contained a signal peptide structure, and the predicted cleavage site was located at amino acids 31–32 (prediction probability 0.867). The CD-search functional domain prediction results showed that the gene contained three characteristic functional domains, CuRO_1_LCC_plant at positions 37-153, CuRO_2_LCC_plant at positions 168-316, and CuRO_3_LCC_plant at positions 429-566. The gene sequence was submitted to the NCBI database at PV341664.

**Figure 1:**
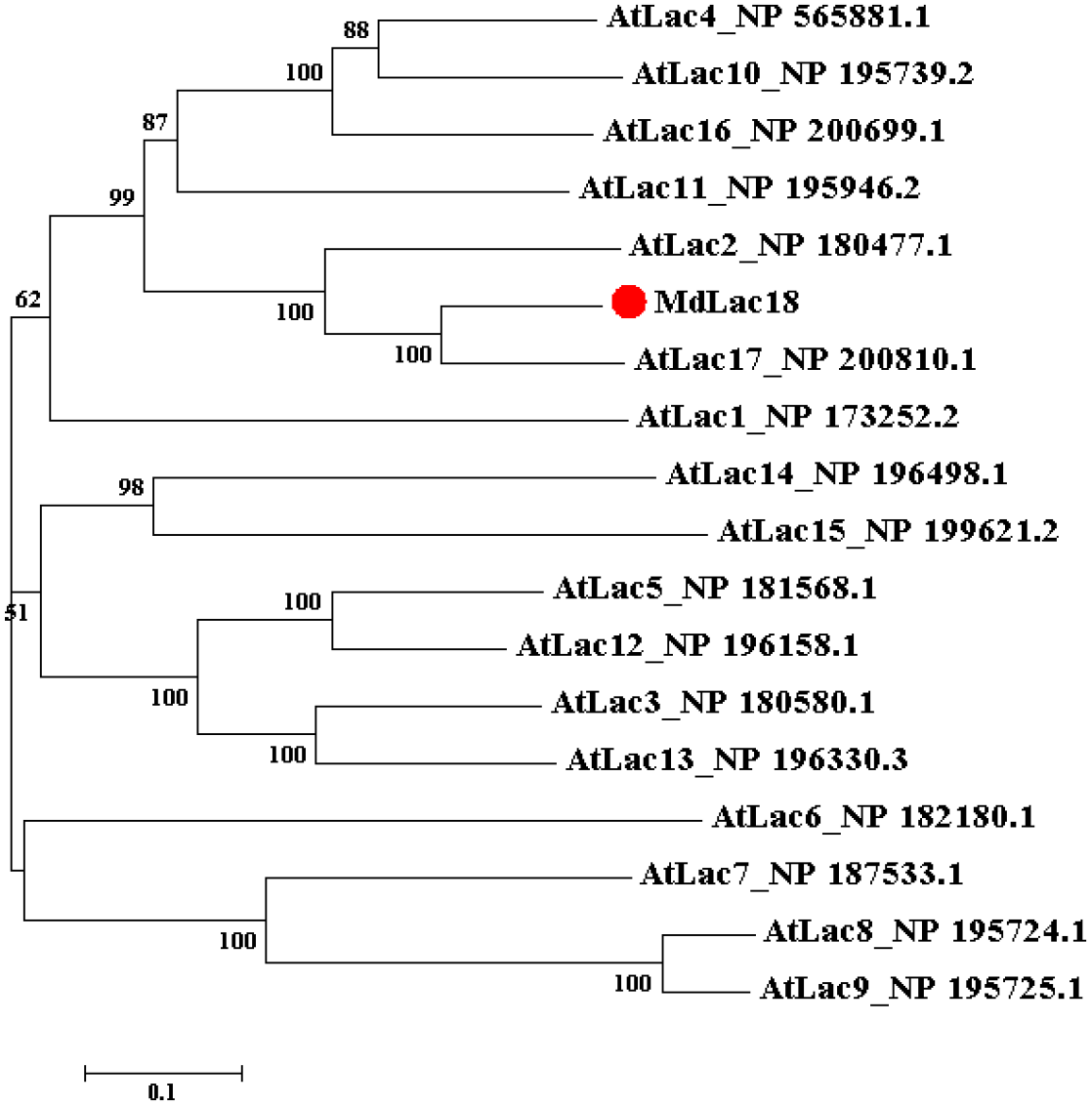
Phylogenetic tree of MdLac18 and 17 laccase genes from *Arabidopsis thaliana*.

Phylogenetic analysis showed that MdLac18 was clustered in the same clade as AtLac17 (**Figure 1**). Previous studies have demonstrated that AtLac17 is closely involved in lignin biosynthesis in Arabidopsis (Berthet et al., 2011). The close evolutionary relationship between MdLac18 and AtLac17 suggests that MdLac18 may play a conserved role in regulating lignin synthesis in apple.

The evolutionary tree was built using the Neighbor-Joining (NJ) method with 1000 bootstrap replicates. Numbers at the nodes indicate the bootstrap support values. The red solid circle indicates the apple laccase protein MdLac18 investigated in this study. The scale bar represents 0.1 amino acid substitutions per site.

### 3.2 Identification and screening of transgenic tobacco

**Figure 2:**
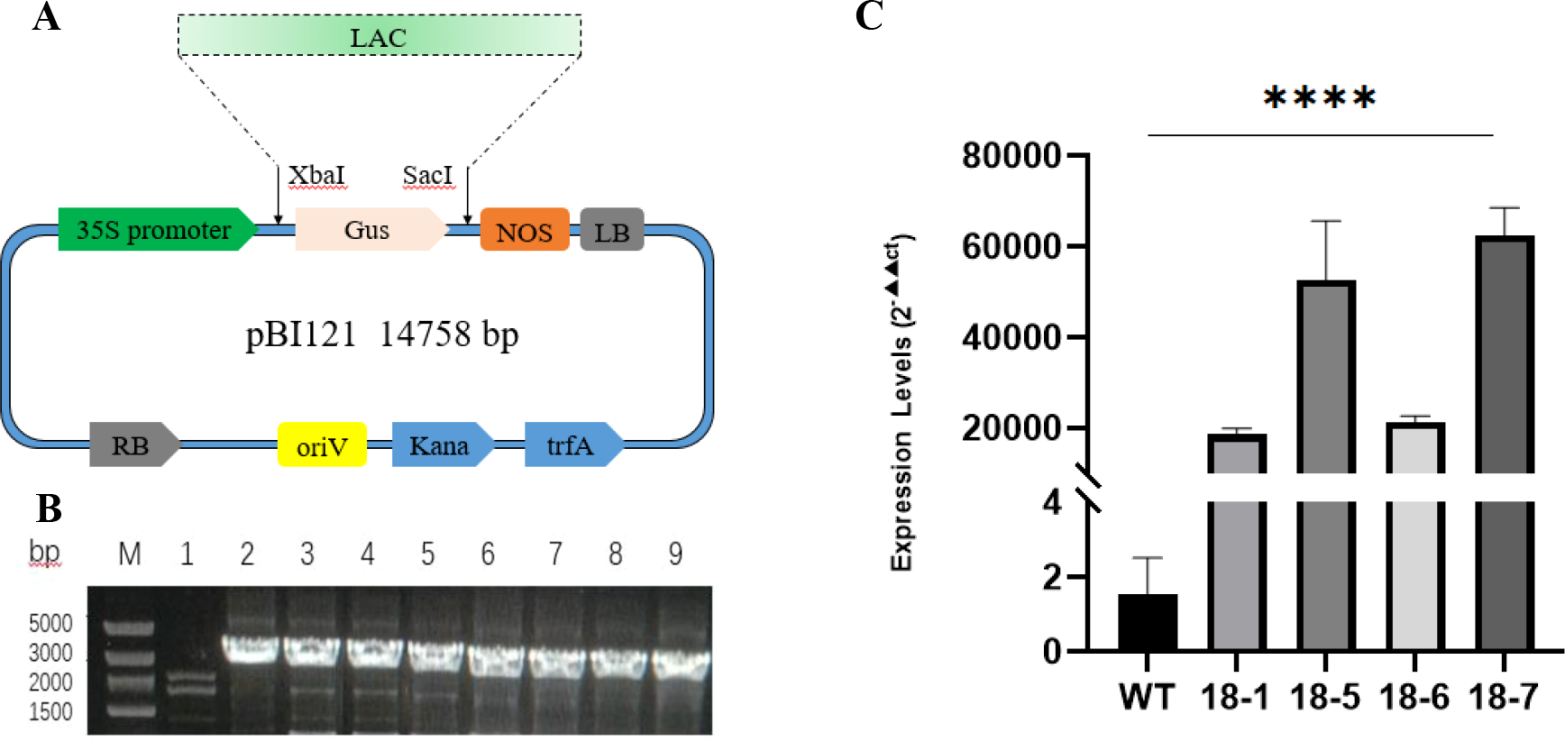
**(A)** Chromatogram of pBI121-*LAC* recombinant vector. **(B)** Identification at the DNA level of the plants transformed with the *Lac18* gene. M. DL5000 Marker; 1. Negative control; 2–9. Identification results of tobacco plants transformed with pBI121-*Lac18*. **(C)** Identification at the RNA level of *Lac18* in transgenic plants. **** indicates a statistically significant difference at p < 0.0001.

PBI121-*MdLac18* was transferred into EHA105 by freeze-thaw method. Through PCR identification, EHA105-pBI121-*MdLac18* was constructed and the bacterial liquid was stored for subsequent experiments (**Figure 2A**).

The tobacco aseptic seedlings (cultured for 45 days) were subjected to pre-culture, agrobacterium infection, dark culture and differentiation screening to obtain regenerated buds and transgenic plants; A total of 8 independent T1 transgenic lines were generated, and the results of DNA level identification showed that 8 MdLac18 positive transgenic plants were obtained (**Figure 2B**).

After one month’s acclimation, total RNA was extracted from the plants, and the expression level of *MdLac18* gene was detected by qPCR. The results showed that the expression levels of the target genes in four transformed *MdLac18* tobacco plants were significantly up-regulated, and the relative expression levels were increased by 18604 times, 52711 times, 21373 times and 62523 times as compared with those in the wild-type (WT) tobacco plants, respectively (**Figure 2C**). The line with stable expression level and consistent phenotypic performance was selected for subsequent detailed trials based on the results of qualitative and quantitative analysis.

### 3.3 Phenotypic observation of transgenic tobacco

**Figure 3:**
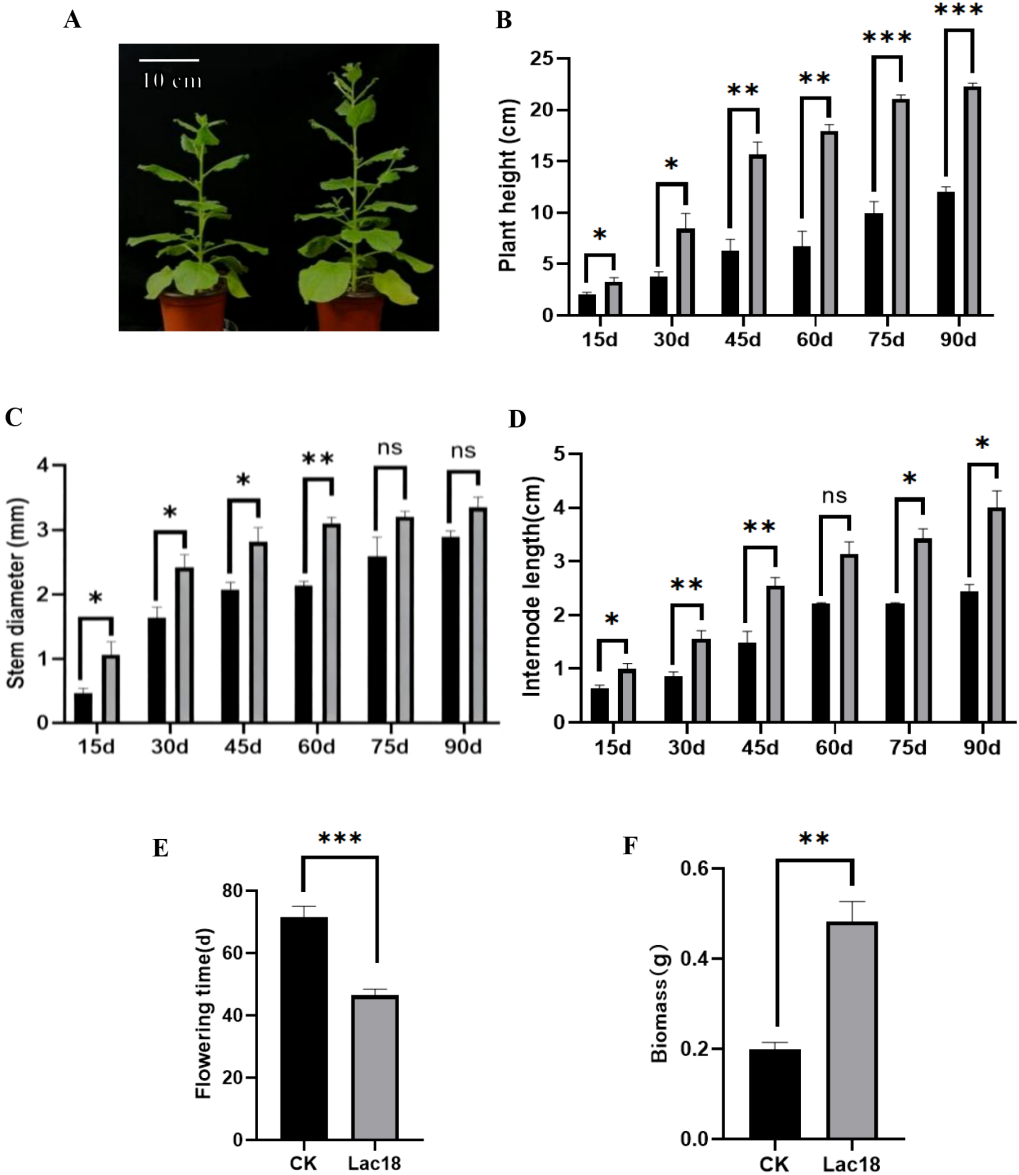
Phenotypic observation of *Lac18* transgenic tobacco and common tobacco. **(A)** 60d growth.On the left of each picture is CK, and on the right is transgenic tobacco. **(B)** Measuring the plant height of the tobacco grown for 90 days, and analyzing the significant difference by t-test; *significant difference at p<0.05 level. **(C)** Dynamic changes of stem diameter between *Lac18* transgenic tobacco and normal tobacco. **(D)** Dynamic changes of internode length between transgenic tobacco with *Lac18* gene and normal tobacco. **(E)** Flowering time of *Lac18* transgenic tobacco and common tobacco. **(F)** Comparison of biomass of *Lac18* transgenic tobacco and normal tobacco. T-test shows significant difference; *, significant difference at p<0.05 level.

After the *MdLac18* gene was transferred into *N. benthamiana* seed, the seeds were collected to obtain a T1 generation; After seeding, positive transgenic lines were screened by PCR and the growth of the line and WT were observed for 90 days and the relevant data were recorded every 15 days. The results showed that the *MdLac18* transgenic lines showed significant growth advantages throughout the growth cycle. The plant height of transgenic tobacco was significantly higher than that of the WT at each time point, and the difference in plant height gradually increased with the growth time. For example, on day 90, the plant height was greater than 20 cm in the *MdLac18* group and about 12 cm in the WT group, a very significant difference (**Figures 3A, B**). This indicated that the overexpression of *MdLac18* gene significantly promoted the longitudinal growth of tobacco plants in a time-dependent manner.

The diameter of tobacco stem base was measured with a vernier caliper (accuracy 0.01 mm). The results showed that the stem diameter of *MdLac18* transgenic tobacco was significantly higher than that of the WT on days 15, 30, 45 and 60. There was no significant difference in stem diameter between the two groups on days 75 and 90 (**Figure 3C**). This indicated that *MdLac18* gene overexpression significantly promoted the stem diameter growth in the early stage of tobacco growth (15–60 days) (transgenic: 4.2 ± 0.3 mm; control: 3.1 ± 0.2 mm, P < 0.01) and and the promotion effect disappeared in the late growth stage (75–90 days), which was attributed to the determinate growth characteristic of *N. benthamiana* (a herbaceous annual plant) with stem diameter reaching a plateau in late stages and the compensation of normal cell elongation in WT plants.

The robust internode in the middle of the plant was selected, and the linear distance between two adjacent internodes was measure. The results showed that the internode lengths of *MdLac18* transgenic tobacco were significantly higher than that of the WT at 15d, 30d, 45d, 75d and 90d, and there was no significant difference only on 60d (**Figure 3D**). This indicated that the overexpression of *MdLac18* gene promoted the internode elongation of tobacco, and the promotion was significant at most growth stages.

Taking the complete unfolding of the first flower petal of a tobacco plant as a flowering judgment standard, and counting the first flowering days of each tobacco plant. The flowering time of *MdLac18* transgenic tobacco was significantly earlier than that of the WT, and the difference was extremely significant (**Figure 3E**). This indicated that overexpression of *MdLac18* gene could significantly promote early flowering of tobacco.

The whole plant was harvested when the tobacco grew to 90 days, dried to constant weight at 80°C, and weighed after cooling to determine the biomass. The results showed that the biomass of tobacco transgenic with *MdLac18* gene was significantly higher than that of the WT, with a significant difference (**Figure 3F**). This indicated that overexpression of *MdLac18* gene could significantly increase tobacco biomass and promote plant material accumulation.

### 3.4 Comparison of EPG indicators of *M. persicae* feeding on laccase-transgenic tobacco plants

From the non-phloem stage of *M. persicae* feeding on tobacco, when *M. persicae* feeding on *MdLac18* transgenic tobacco plants, the times of probing, the time of first starting probing, the total time of np wave, the total time of G wave and the times of pd wave were significantly higher than those of common tobacco, and the total time of C wave was not significantly different between the two tobaccos (**Table 3**).

**Table 3:** Comparison of EPG parameters of *M. persicae* feeding on laccase-transgenic tobacco plants.

EPG parameter	Variety	
	WT tobacco	Transgenic tobacco (*MdLac18*)
Probing times	1.50 ± 1.00	4.00 ± 1.41*
First probing time (s)	0.64 ± 0.28	4.92 ± 2.77*
Np wave total time (s)	0.71 ± 0.29	199.87 ± 25.52*
C wave total time (s)	18676.60 ± 2726.07	19200.87 ± 3412.75
G wave total time (s)	203.22 ± 122.01	2143.12 ± 461.09*
Pd wave times	40.00 ± 14.09	80.17 ± 15.56*
E1 wave total time (s)	58.25 ± 34.67	688.45 ± 143.62*
E2 wave total time (s)	593.70 ± 158.12	147.71 ± 10.21*
E1 times	4.67 ± 2.52	1.50 ± 1.00
E2 times	1.67 ± 1.21	1.00 ± 0.63
E1 proportion to total wave type (%)	0.33 ± 0.17	2.75 ± 1.21*
Proportion E2 to total wave type (%)	2.75 ± 0.73	0.46 ± 0.24*
Time to first reach phloem (s)	885.44 ± 507.60	18470.53 ± 4849.25*
Time (s) for the first occurrence of E2 wave	2661.48 ± 1698.62	10084.10 ± 2121.54*
E2>10min(%)	83.33 ± 28.87	0.00 ± 0.00*

The data in the table are the average standard errors. * after peer data indicates that the difference is significant at the P < 0.05 level by independent sample t-test ± standard errors.

From the phloem stage of *M. persicae* feeding on tobacco, the total time of E1 wave, the proportion of E1 to the total wave pattern, the time for the first time to reach the phloem and the time for the first occurrence of E2 wave when *M. persicae* feeding on *MdLac18* transgenic tobacco plants were significantly higher than those of normal tobacco. The total E2 wave time, the proportion of E2 to the total wave pattern, and the proportion of E2>10 min were all significantly lower than those of common tobacco (**Table 3**).

**Figure 4:**
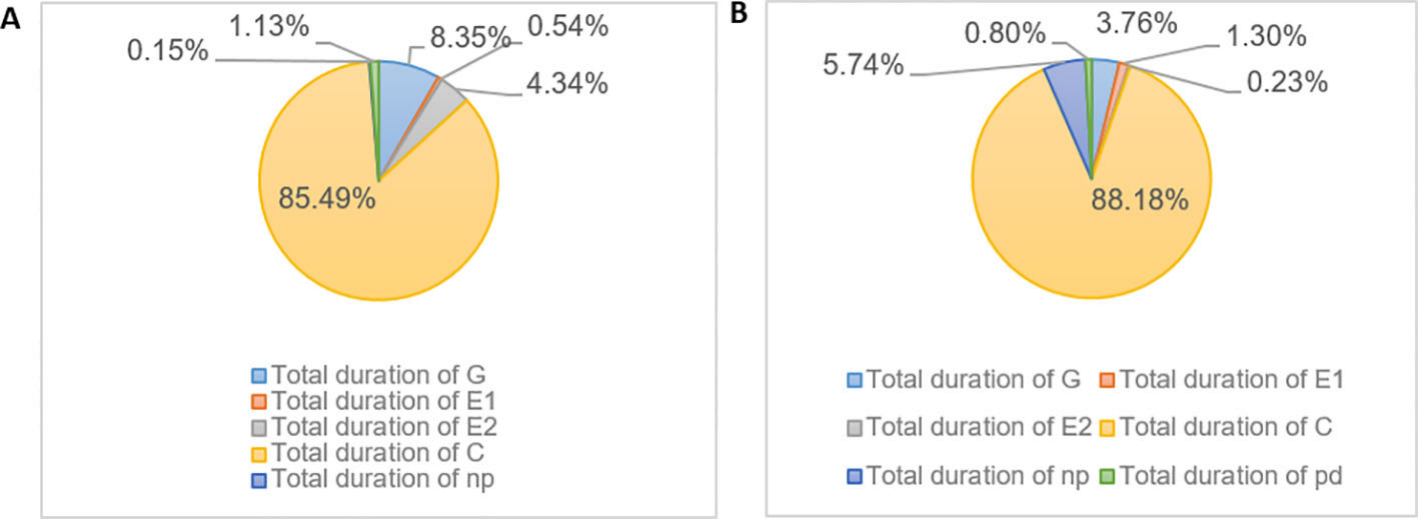
Percentage of the average duration of various waveforms of *M. persicae* feeding for 6 hours on common tobacco **(A)** and tobacco transformed with the *Lac18* gene **(B)**.

The time proportion of *M. persicae* feeding on *MdLac18* transgenic tobacco and normal tobacco was analyzed in each wave mode. The results showed that when *M. persicae* feeding on *MdLac18* transgenic tobacco, the total time of G wave accounted for 3.76%, which was lower than that of normal tobacco (8.35%). The total time of E1 wave (salivation in phloem) accounted for 1.30%, which was higher than that of E1 wave in common tobacco (0.54%). The total time of E2 wave (feeding in phloem) was 0.23%, which was lower than that of normal tobacco (4.34%). The time proportion of E wave (E1+E2) was 1.53%, which was lower than the total time proportion of E wave in common tobacco (4.88%); The above data indicated that *M. persicae* needed to spend more time to find the appropriate feeding site on transgenic tobacco. After finding the appropriate site, it would take a long time to secrete saliva and need to secrete more saliva to digest food, but the feeding time was short, suggesting that transgenic laccase tobacco was not conducive to *M. persicae* feeding (**Figure 4**).

### 3.5 Effects of transgenic tobacco with laccase gene on the survival and reproduction of *M. persicae*

**Figure 5:**
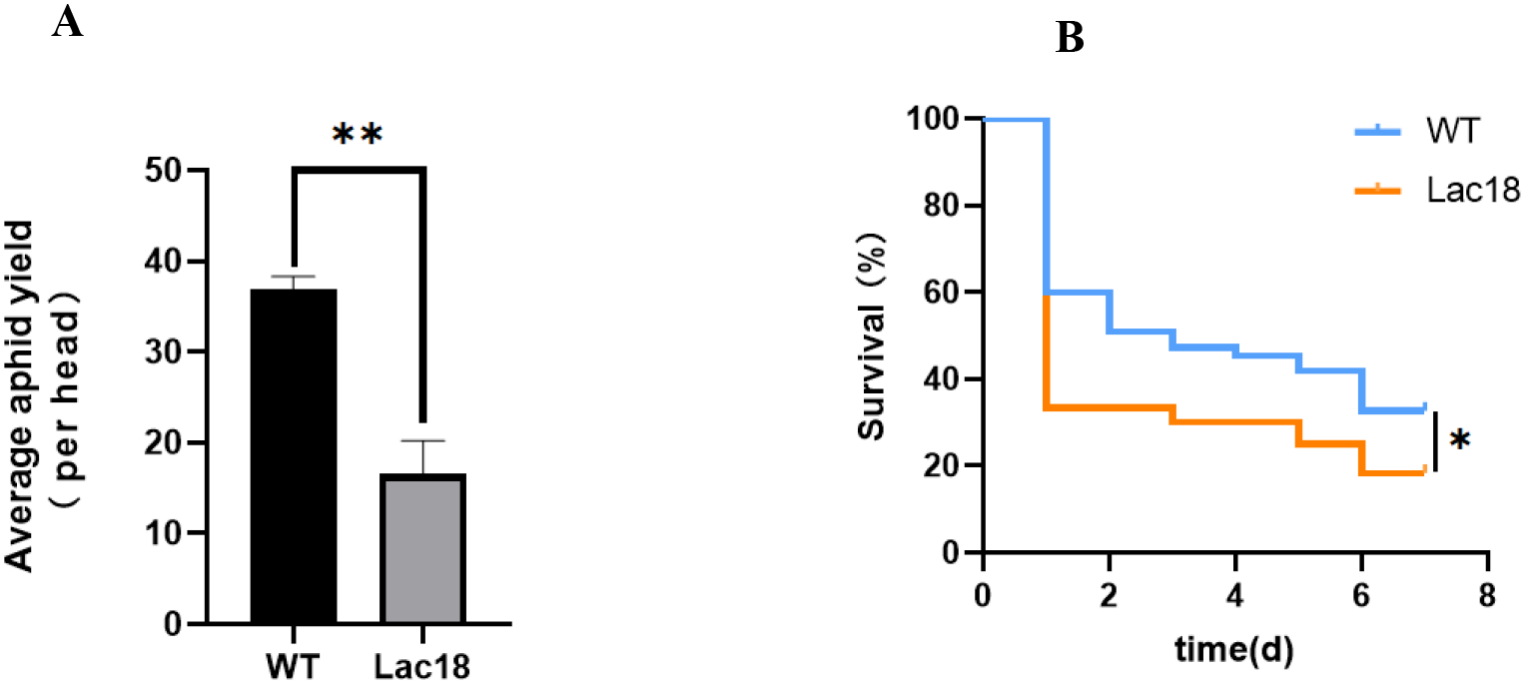
**(A)** Survival rate and **(B)** aphid yield of *M. persicae* in *Lac18* transgenic tobacco. * p < 0.05, ** p < 0.01.

As shown in **Figure 5**, on the 8th day, the survival rate of *M. persicae* on *MdLac18* transgenic tobacco (18.33%) was significantly lower than that of normal tobacco (32.73%); In terms of reproductive indexes, the average aphid yield of *M. persicae* on *MdLac18* transgenic tobacco (16.57 heads) was significantly lower than that of the control group (36.93 heads). The above results indicated that overexpression of *MdLac18* gene could significantly reduce the survival rate of *M. persicae* and inhibit its average reproductive ability.

### 3.6 Physiological and biochemical analysis of transgenic tobacco

**Figure 6:**
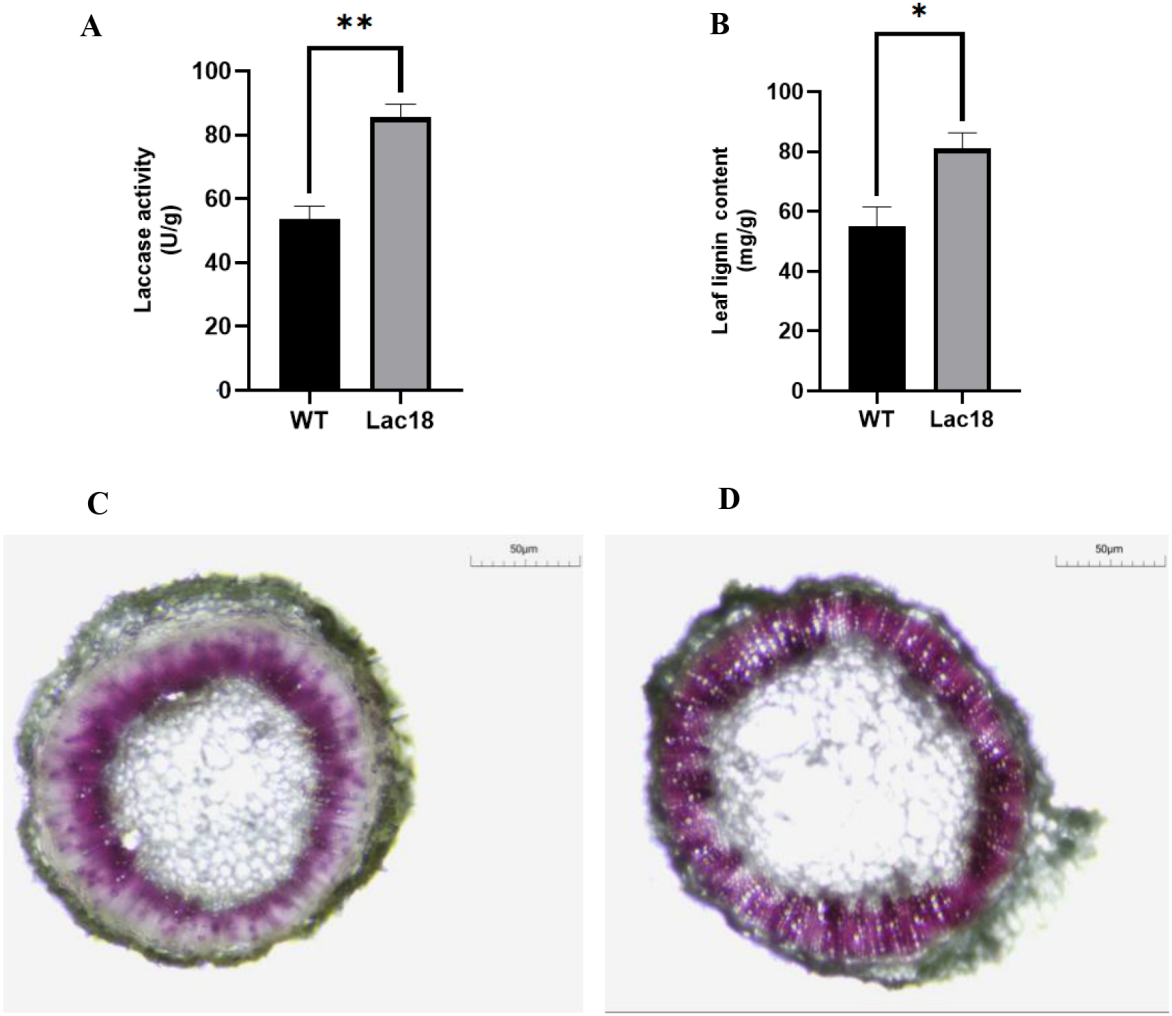
**(A)** Detection of laccase activity in transgenic tobacco. **(B)** Determination of the lignin content in transgenic tobacco.The significance of differences was analyzed by t-test; *: Significantly different at the p<0.05 level. **(C, D)** Phloroglucinol-HCl staining of stem cross-sections (bar = 50 μm); **(C)** WT tobacco; **(D)** *Lac18*-overexpressing tobacco. Purple color indicates lignin accumulation. * and ** indicate statistically significant differences at p < 0.05 and p < 0.01, respectively.

**Figure 6**. A shows that the average laccase activity of tobacco is about 53.70 U/g. After the *MdLac18* gene was transferred, the average laccase activity of tobacco was increased to about 85.71 U/g, and compared with the WT, the overall laccase activity in transgenic tobacco was increased by about 59.61%.

The lignin content of *MdLac18* transgenic tobacco and WT leaves was detected quantitatively by acetylation. As shown in **Figure 5B**, the average lignin content in WT leaves was 54.92 mg/g, while the average lignin content in leaves of *MdLac18* transgenic plants was significantly increased to 80.95 mg/g, representing an increase of 47.40% as compared with that in WT. The above results indicated that overexpression of *MdLac18* gene could significantly promote lignin biosynthesis in the tobacco leaves.

In order to further explore the function of *MdLac18* gene, 8-week-old tobacco transgenic with *MdLac18* gene and WT ipsilateral stem segments were selected, cut into slices, stained with phloroglucinol, and the histological structure of lignin was observed under the microscope. As shown in **Figures 5C, D**, the lignin staining of WT (left)stalk was light reddish purple, while that of *MdLac18* transgenic plants(right) was deeper, and the color-developing area was significantly enlarged, especially in the vascular tissue. To sum up, compared with WT, the lignin content of *MdLac18* transgenic tobacco was significantly increased, and the vascular tissue was more developed.

### 3.7 Analysis of lignin monomer composition in *Lac18*-overexpressing transgenic tobacco plants

To elucidate the regulatory role of the *MdLac18* gene in lignin monomer biosynthesis in tobacco, thioglycolic acid hydrolysis (for cleavage of lignin ether linkages) coupled with gas chromatography-mass spectrometry (GC-MS) was utilized to quantify the relative contents of lignin monomers in *Lac18*-overexpressing transgenic tobacco lines and wild-type (WT) plants. Monomer structures were identified as coniferyl alcohol derivatives via library searching using NIST MS Search v.2.3 software, and coniferyl alcohol is a well-characterized precursor for the synthesis of G-type lignin monomers.

Quantitative analysis revealed that the average relative peak area of coniferyl alcohol derivatives in WT lines was (13.1 ± 7.7)×10^4^, whereas that in *Lac18*-overexpressing transgenic lines reached (36.1 ± 8.8)×10^4^, corresponding to a 176% significant increase compared with WT (*p<0.05). Collectively, these results indicate that *Lac18* overexpression markedly enhances the relative abundance of G-type lignin monomers in transgenic tobacco, demonstrating that the *Lac18* gene can specifically and efficiently promote G-type lignin monomer biosynthesis in tobacco.

**Figure 7:**
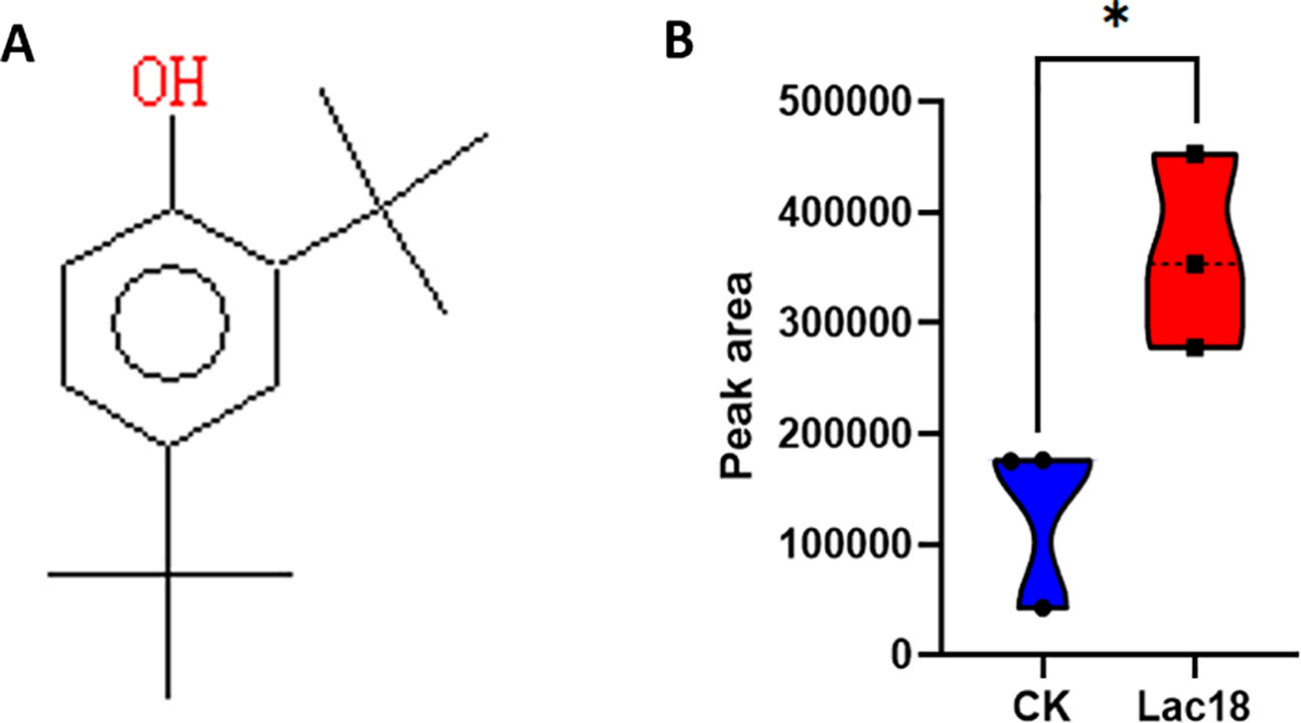
Detection of lignin monomer composition and comparison of relative peak areas in *Lac18*-overexpressing transgenic tobacco. **(A)** Coniferyl alcohol derivative; **(B)** Comparison of relative peak areas of coniferyl alcohol derivatives at retention time (RT) = 6.564 min between wild-type (CK) and *Lac18*-overexpressing transgenic tobacco plants. * indicates a statistically significant difference at p < 0.05.

We further hypothesize that the aphid resistance conferred by *Lac18* is primarily mediated by the aphid-inhibitory activity of G-type lignin synthesized under its catalytic regulation. As a core functional constituent of the tobacco cell wall, the accumulation of such specific monomers directly modulates the mechanical robustness of the cell wall and contributes to aphid resistance-related phenotypic traits (**Figure 7**).

## 4 Discussion

### 4.1 *MdLac18* acts as a dual-functional regulator coordinating aphid resistance and plant growth

Laccase genes are well recognized for their involvement in plant secondary metabolism and stress responses, but their application in engineering aphid resistance—especially in non-model perennial woody crops like apple—remains inadequately explored (Ding et al., 2025). This study provides the first comprehensive characterization of the apple-derived *MdLac18* gene, establishing it as a rare dual-functional regulator that synergistically enhances aphid resistance and plant growth in a heterologous expression system (*N. benthamiana*).

Unlike previous studies focusing on laccase-mediated disease resistance or growth regulation in model plants (Zhang et al., 2019; Wang et al., 2020), our findings demonstrate that *MdLac18* confers robust resistance against *M. persicae* while significantly improving key agronomic traits. This cross-species validation (apple → tobacco) not only underscores the evolutionary conservation of *MdLac18*’s core functions but also highlights its potential as a versatile genetic tool for broad-spectrum crop improvement. Importantly, this work fills the critical gap in utilizing woody plant laccase genes to address both pest resistance and yield maintenance, which has long been a bottleneck in agricultural biotechnology (Yang et al., 2024).

### 4.2 Lignin-mediated physical barrier: the core mechanism of aphid resistance

Our physiological, behavioral, and histological analyses collectively elucidate that MdLac18-mediated aphid resistance operates primarily through a lignin-dependent physical barrier mechanism (Hao et al., 2024; Li et al., 2025). Consistent with the conserved role of laccase as the rate-limiting enzyme in lignin polymerization (Wang et al., 2017; Qin et al., 2020), heterologous overexpression of MdLac18 in Nicotiana benthamiana significantly increased laccase activity by 59.61% and total lignin content by 47.40%, accompanied by pronounced lignification of stem vascular tissues. This structural reinforcement directly impeded the feeding behavior of Myzus persicae, as evidenced by comprehensive Electrical Penetration Graph (EPG) assays.

Aphids colonizing MdLac18-overexpressing plants exhibited drastically prolonged salivation phases (E1 wave) and shortened phloem ingestion durations (E2 wave), with no individuals achieving sustained phloem feeding (E2 > 10 min). These distinct behavioral shifts indicate that the thickened, lignin-reinforced cell walls formed a formidable barrier that prevented efficient stylet penetration into the vascular tissue. Consequently, aphids were forced to expend excessive energy on exploratory probing and salivation without successful nutrient acquisition, leading to a 43.99% increase in corrected mortality and a 55.13% reduction in fecundity by the 8th day post-inoculation.

Notably, thioglycolic acid hydrolysis coupled with gas chromatography-mass spectrometry (GC-MS) further revealed the substrate specificity of MdLac18 in modulating lignin monomer composition: the relative abundance of G-type lignin monomers (coniferyl alcohol derivatives) in transgenic lines was elevated by 176% compared with wild-type plants. This finding aligns with previous reports demonstrating that G-type lignin contributes more substantially to cell wall mechanical rigidity than S-type or H-type lignin monomers (Nancy et al., 2020; Qin et al., 2020). Unlike the more flexible S-type lignin that features fewer interunit linkages, G-type lignin forms a densely cross-linked, rigid network that dramatically enhances the physical strength of the secondary cell wall. The MdLac18-driven enrichment of G-type lignin thus represents a targeted regulatory strategy to optimize the defensive efficacy of the lignin barrier, rather than a nonspecific increase in total lignin content.

This mechanism aligns with prior studies linking lignin accumulation to enhanced resistance against piercing-sucking pests in herbaceous plants (An et al., 2019; Hu et al., 2018) but extends beyond them by demonstrating the specific contribution of a woody plant-derived laccase to this process, as well as the critical role of G-type lignin monomer specialization in reinforcing defense capacity. The synergistic effects of increased total lignin content and G-type lignin enrichment likely act in concert to strengthen cell wall rigidity, creating a dual-layered physical barrier that effectively blocks aphid stylet penetration and suppresses pest colonization.

### 4.3 Breaking the growth-defense trade-off: a rare “dual-benefit” phenotype

Notably, wild-type (WT) control Nicotiana benthamiana plants showed growth retardation, with a height of ~12 cm at 90 days, significantly lower than their normal growth level in the field or standard greenhouses. This was not caused by differences in inter-group culture conditions, but may be related to environmental factors such as light, soil, water, humidity and ventilation in the growth chamber. These factors indicate that the experimental environment was a suboptimal growth environment for N. benthamiana. Since both transgenic and wild-type plants were cultured under the same conditions (25 °C light incubator), the two groups had strict comparability; thus, the growth advantage of transgenic plants should be interpreted as improved stress tolerance rather than growth promotion under normal conditions. This improved stress tolerance enables transgenic plants to better adapt to suboptimal environments, reduce growth inhibition, and perform better, which is consistent with the stress regulatory role of the target gene and also supports its potential in improving plant adaptability to adverse environments.

One of the most striking findings of this study is that *MdLac18* overexpression defies the classical “growth-defense trade-off” paradigm, a core bottleneck in plant breeding that arises from the energetic and metabolic competition between defense activation and growth-related processes. Typically, constitutive activation of defense pathways (e.g., lignin biosynthesis) consumes substantial metabolic resources that would otherwise support growth and development, leading to growth retardation and yield penalties (Huot et al., 2014). In contrast, *MdLac18*-overexpressing tobacco lines exhibited a distinctive “dual-benefit” phenotype: superior aphid resistance alongside significant improvements in key agronomic traits, including plant height, stem diameter, internode length, early flowering, and biomass accumulation.

To interpret this unique phenotypic pattern, we propose two non-mutually exclusive hypothetical mechanisms based on our phenotypic observations and previous published studies, which await further experimental validation. First, enhanced lignification of xylem vessels in transgenic lines may potentially improve hydraulic conductivity and mechanical structural support (Ménard et al., 2022). The reinforced xylem structure could facilitate the transport of water and nutrients to aerial tissues, which may provide a structural basis for robust vegetative growth of transgenic plants. Second, accumulating evidence has shown that laccases can interact with auxin signaling pathways in multiple plant species (Qin et al., 2020), and this interaction may fine-tune hormone homeostasis to promote cell elongation and tissue expansion. It is plausible that MdLac18 may exert a similar regulatory effect on auxin signaling, which could contribute to the promoted plant growth observed in our study. Collectively, these hypothetical regulatory modes suggest that MdLac18 may optimize plant growth and defense responses simultaneously by improving structural reinforcement and resource allocation efficiency. This unique regulatory characteristic enables MdLac18 to circumvent the metabolic trade-offs commonly associated with defense-related genes, which is particularly valuable for crop improvement as it addresses the long-standing challenge of reconciling pest resistance with yield stability. The potential regulatory links between MdLac18 and hydraulic conductivity, as well as the specific interaction mechanism between MdLac18 and auxin signaling pathways, will be the focus of our in-depth experimental investigation in future research.

**Figure 8:**
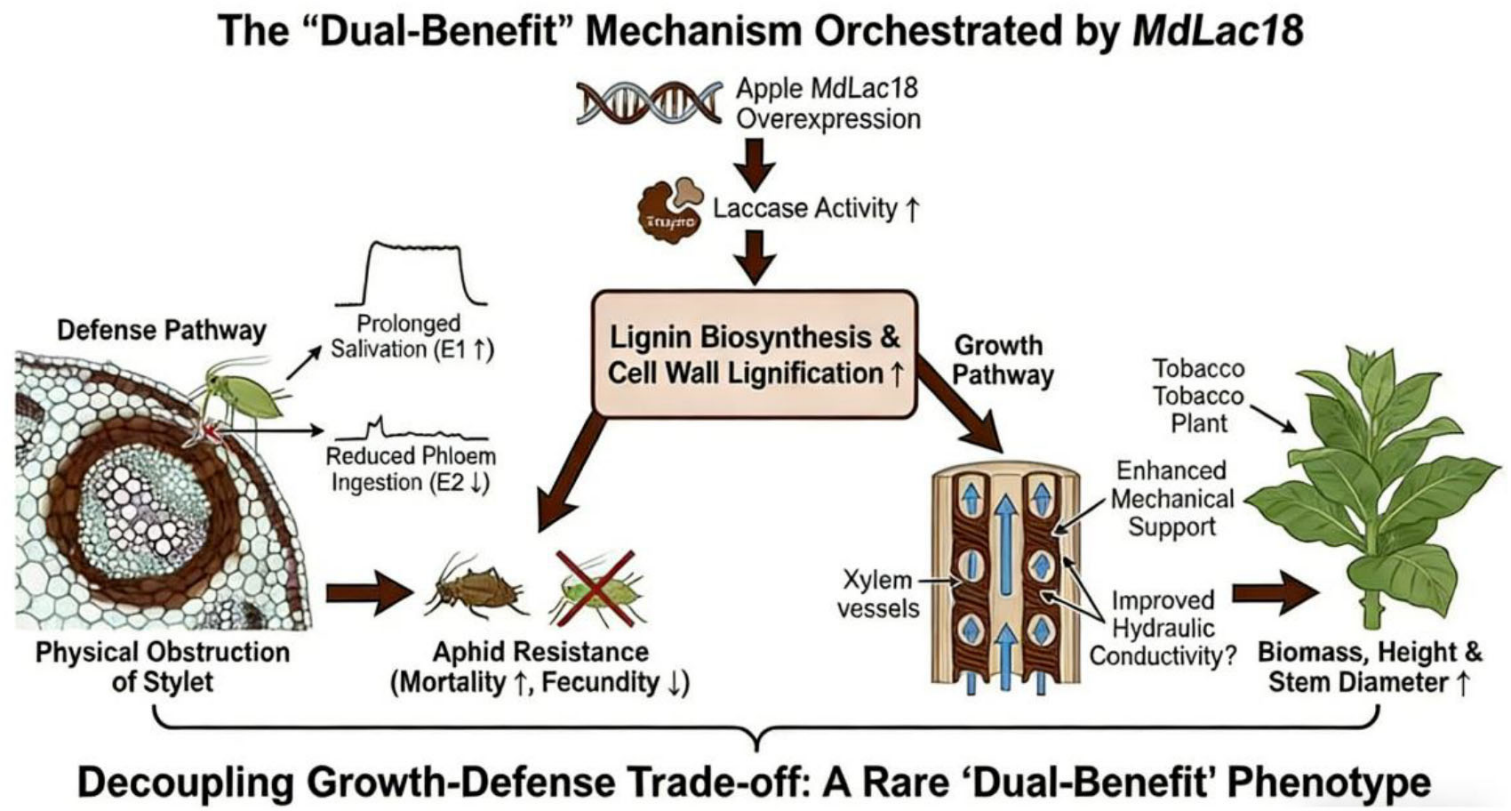
Schematic model of the dual-functional mechanism mediated by MdLac18 in coordinating aphid resistance and plant growth enhancement. * indicates a statistically significant difference at p < 0.05.

This ability to decouple defense activation from growth inhibition makes *MdLac18* an exceptionally valuable genetic resource. Unlike previously reported laccase genes that primarily regulate either resistance or growth (Wang et al., 2020; Wei et al., 2021), *MdLac18* integrates both functions, offering a novel solution to the growth-defense conundrum that has plagued agricultural biotechnology. To visually synthesize the core regulatory logic of *MdLac18*-mediated dual functions, we constructed a mechanistic model (**Figure 8**) that systematically illustrates the cascade of events triggered by this apple-derived gene in the heterologous tobacco system.

All transgenic and WT plants in this study were cultured under the same suboptimal growth conditions for Nicotiana benthamiana, which imposed mild environmental stress and led to significant growth retardation of WT plants. The concurrent increase in lignin content and biomass in MdLac18-overexpressing lines was not a simple growth promotion under resource-sufficient conditions, but a reflection of the stress regulatory function of MdLac18—this gene enhanced plant adaptability to suboptimal environments, thus alleviating stress-induced growth inhibition while activating lignin-mediated aphid resistance.

### 4.4 Implications for sustainable crop breeding and future directions

The successful heterologous expression of *MdLac18* and its dual-functional performance in tobacco demonstrate a feasible strategy for interspecific utilization of woody plant genetic resources. By enhancing the plant’s intrinsic physical defense barrier, *MdLac18* reduces reliance on chemical pesticides, aligning with the goals of sustainable and green agriculture (Guo et al., 2022). This is particularly relevant for apple and tobacco production, where aphid infestations cause substantial economic losses and environmental risks associated with pesticide use (Shah et al., 2025);.

Future work should focus on three key directions to expand the translational value of this research. First, validating the function of *MdLac18* in apple germplasm will be critical to confirm its utility in native host species and support apple breeding programs. Second, exploring the molecular interplay between *MdLac18* and growth-regulating pathways (e.g., auxin signaling, carbon metabolism) will deepen our understanding of how it breaks the growth-defense trade-off. Third, combining *MdLac18* with other resistance genes could develop multi-resistant crop varieties, further enhancing pest management efficacy.

In conclusion, *MdLac18* represents a candidate gene of high agricultural value, offering a promising solution for breeding crops that are both resilient to piercing-sucking pests and agronomically superior. Our findings not only advance the understanding of laccase-mediated dual functions in plants but also provide a technical framework for utilizing woody plant genetic resources to address critical challenges in sustainable agriculture.

### 4.5 Potential artifacts of heterologous overexpression

We recognize the extremely high relative expression of MdLac18 in transgenic N. benthamiana (18604–62523-fold vs. wild-type), which may raise concerns about overexpression artifacts such as non-specific stress responses or metabolic imbalances that do not reflect its natural function. This extreme relative expression results from the absence of endogenous MdLac18 in wild-type N. benthamiana (zero baseline)—a common phenomenon in heterologous expression of exogenous genes in non-homologous plant species, and it does not directly mean an excessively high absolute expression of the exogenous protein in transgenic lines.

To assess the risk of non-specific effects, we comprehensively analyzed phenotypic and physiological data. First, four independent high-expression T1 lines showed specific and consistent dual phenotypes: enhanced aphid resistance and improved agronomic traits (increased plant height, stem diameter, biomass and early flowering), with no typical stress symptoms (e.g., growth retardation, leaf yellowing, wilting). Non-specific stress or metabolic imbalances would likely cause random, inconsistent abnormal phenotypes instead of this uniform positive trait pattern. Second, the phenotypic and physiological changes were specifically associated with the conserved laccase function of MdLac18: its overexpression led to a 59.61% increase in laccase activity, 47.40% rise in lignin content, 176% enrichment of G-type lignin monomers and enhanced vascular lignification (Ishida et al., 2024; Qin et al., 2020), forming a complete gene function-phenotype correlation chain—lignin-mediated physical barrier for aphid resistance and improved vascular structural support for growth promotion. No non-specific metabolic disorder or stress response was found in physiological and biochemical assays.

Published studies further confirm the specificity of our observations: the laccase-regulated lignin biosynthesis pathway is highly conserved in angiosperms (Xie et al., 2020; Liu et al., 2024), so overexpressing woody plant laccase genes in herbaceous plants rarely triggers non-specific stress, only inducing the specific accumulation of lignin and its precursors without global metabolic disorder. For example, Hu et al. (2018) and Nancy et al. (2020) reported that overexpressing GhLac1 (cotton) and TaLAC4 (wheat) enhanced pest and disease resistance via lignin regulation, with no non-specific metabolic imbalances or abnormal stress phenotypes observed.

Although the above evidence rules out overexpression artifacts, this extreme relative expression has limitations: it cannot fully reflect the native expression pattern and regulatory network of MdLac18 in Malus domestica. In follow-up research, we will conduct stable, near-endogenous expression of MdLac18 in apple combined with gene knockout/knockdown technology, to further verify its natural function and regulatory mechanism in apple aphid resistance and growth, thus providing more direct genetic evidence for its breeding application.

## 5 Conclusion

This study systematically characterized the laccase gene *MdLac18* from the aphid-resistant apple cultivar ‘Starkrimson’ and validated its dual functions in enhancing aphid resistance and promoting plant growth through heterologous expression in *N. benthamiana*(4 independent T1 transgenic lines generated, 100% phenotypic frequency for growth promotion and aphid resistance). The key findings are summarized as follows:

First, MdLac18 overexpression confers robust resistance to M. persicae in transgenic tobacco. Phenotypic assays demonstrated that the corrected aphid mortality rate reached 43.99% and fecundity was reduced by 55.13% on the 8th day post-inoculation. EPG analysis further revealed that the resistance mechanism is associated with prolonged aphid salivation (E1 wave) and shortened phloem ingestion (E2 wave), which is attributed to MdLac18-mediated lignin accumulation (47.40% increase), enhanced vascular tissue lignification and specific promotion of G-type lignin biosynthesis (176% increase in G-type monomers, reduced S/G ratio). Histological staining confirmed enhanced lignification in stem vascular tissues, forming a physical barrier that obstructs aphid stylet penetration—highlighting MdLac18 as the first apple-derived laccase gene with confirmed aphid-resistant function.

Second, MdLac18 exhibits the potential to decouple the classical “growth-defense trade-off” paradigm under controlled suboptimal conditions by exerting its stress regulatory function, a major bottleneck in crop breeding. Unlike typical defense-related genes that impair plant growth, MdLac18 overexpression significantly improved key agronomic traits, including plant height, stem diameter at the early vegetative stage (30–60 days), internode length, early flowering, and biomass accumulation. This “dual-benefit” phenotype is likely driven by optimized structural support (reinforced xylem vessels) and resource allocation, as inferred from the enhanced lignification and growth promotion observed. Our findings establish MdLac18 as a rare genetic resource with the potential to decouple growth and defense, and its stress regulatory function endows plants with improved adaptability to adverse environments, providing a novel strategy for breeding crops with durable aphid resistance and excellent agronomic performance.

Third, the successful cross-species expression of *MdLac18* (apple → tobacco) validates its potential for interspecific utilization, providing a feasible technical strategy for tapping into woody plant genetic resources for crop improvement. The gene’s ability to simultaneously enhance pest resistance and agronomic performance addresses the critical need for sustainable pest management in agriculture.

In conclusion, *MdLac18* represents a rare and valuable genetic resource that integrates aphid resistance and growth promotion through lignin-mediated cell wall reinforcement. Our findings not only deepen the understanding of laccase-mediated dual regulation in plants but also provide a novel candidate gene and technical framework for breeding high-yield, pest-resistant crops. This work contributes to reducing chemical pesticide use and advancing sustainable agricultural development, with broad implications for apple, tobacco, and other crop breeding programs.

## Data Availability

The original contributions presented in the study are included in the article/supplementary material. Further inquiries can be directed to the corresponding author.
